# The Length of Working Life in Spain: Levels, Recent Trends, and the Impact of the Financial Crisis

**DOI:** 10.1007/s10680-017-9458-9

**Published:** 2018-01-03

**Authors:** Christian Dudel, María Andrée López Gómez, Fernando G. Benavides, Mikko Myrskylä

**Affiliations:** 1 Max Planck Institute for Demographic Research, Konrad-Zuse-Str. 1, 18057 Rostock, Germany; 2 Department of Social and Behavioral Sciences, Harvard T.H. Chan School of Public Health, Boston, MA USA; 3 Center for Research in Occupational Health, Universitat Pompeu Fabra, Barcelona, Spain; 4 CIBER of Epidemiology and Public Health, Barcelona, Spain; 5 IMIM Parc Salut Mar, Social Epidemiology and Occupational Health Group, Barcelona, Spain; 6 Department of Social Policy, London School of Economics and Political Science, London, UK; 7 Population Research Unit, University of Helsinki, Helsinki, Finland

**Keywords:** Great Recession, Length of working life, Multistate life table, Spain, Sullivan’s method, Working life expectancy

## Abstract

While there has been considerable debate about extending the length of working life, relatively little is known about this issue. We use data from the Spanish Continuous Working Life Sample for 2004–2013 to calculate period working life tables, which in turn allows us to assess the impact of the financial crisis on working life expectancy in Spain. Before the recession hit, working life expectancy in Spain was around 38 years for males and 33 years for females. The recession had a tremendous impact on the Spanish labor market, but the effects differed considerably by gender and occupational category. Men working in skilled non-manual jobs were less affected, while men working in unskilled manual jobs lost close to 14 years of working life expectancy. Women were less affected than men. With working life expectancy decreasing, the average proportion of lifetime spent in unemployment and outside the labor market increased markedly, whereas the average number of years spent in retirement changed only a little. When we decompose losses in working life expectancy by age group, we find that economic fluctuations affect both older and younger workers. This result suggests that policies that focus on retirement ages only are incomplete. We also compare our findings to the results obtained by Sullivans method, which is based on prevalence rates rather than the incidence-based working life table approach. We find that the use of Sullivans approach does not accurately reflect the levels of and the trends in working life expectancy.

**Electronic supplementary material:**

The online version of this article (10.1007/s10680-017-9458-9) contains supplementary material, which is available to authorized users.

## Introduction

Population aging will put a strain on social security systems in developed countries in the near future. Increasing the length of working life is widely considered to be a potential remedy to this problem, and this strategy has been advocated by the European Commission (2010). As a consequence, the statutory retirement age has been raised in several countries (OECD 2015). However, more research is needed on the expected length of working life, including on the questions of how working life expectancy (WLE) developed in the past, which factors influence WLE, and how WLE differs by country and region (Dudel and Myrskylä 2016).

Spain is among the countries for which only limited information on WLE is currently available. The financial crisis that started in 2008 had a tremendous impact on the Spanish economy, triggering a severe recession that is now commonly referred to as the “Great Recession” (for a general overview see Jimeno and Santos 2014). According to Eurostat, the unemployment rate in Spain more than tripled between 2007 and 2012, from 8.2 to 24.8%. Young people have been especially vulnerable to the consequences of the crisis, and youth unemployment in Spain has risen considerably (Dolado et al. 2013). The long-term youth unemployment level has also increased, but is low compared to the overall level of unemployment, and compared to the levels in other European countries (O’Higgins 2012).

The potential effects of the crisis on the length of working life have not yet been explored. While we can expect to find that the average length of working life decreased due to unemployment, it is not clear how the losses in working life expectancy are distributed across working ages. For example, high levels of youth unemployment might be associated with losses in WLE at relatively young ages. On the other hand, low levels of long-term youth unemployment could imply that relatively small losses at younger ages come at the cost of increasing job instability, i.e., repeated losses across the working life.

The only estimates of WLE available for Spain are provided by Eurostat and are based on Sullivan’s method (Sullivan 1971). For instance, Eurostat reported that in 2014, the expected duration of working life at age 15 was 37.1 years for Spanish males and 32.3 years for Spanish females. Widely used in health research (e.g., Crimmins and Saito 2001), Sullivan’s method generally combines survival probabilities obtained from life tables with prevalence rates to estimate the time spent in a state of interest. In its estimates of working life expectancy, Eurostat uses labor force participation rates as prevalence rates (Hytti and Nio 2004), including the time spent in both employment and unemployment over the working life.

Unlike Eurostat, most of the scientific studies on WLE cited above used Markov chains based on incidence rates that capture transitions between states. It is well known that Sullivan’s method can lead to biased estimates when mortality rates or incidence rates change over time, as prevalence rates may not adequately capture rapid changes in incidence rates (Mathers and Robine 1997; Newman 1988). It may be expected that the incidence of both (un)employment and labor force participation changed rapidly during the financial crisis. Despite the financial crisis, Eurostat’s recent estimates of the length of working life have been relatively stable, declining slightly from 38.3 years in 2008 to 37.1 years in 2014 for males, and increasing from 29.8 years in 2008 to 32.3 years in 2014 for females.

In this paper, we present estimates of the length of working life, or of working life expectancy, for Spain for the period of 2004–2013. WLE is defined as the expected time spent in employment over a lifetime. We calculate period multistate life tables based on Markov chains, estimated using a large sample from the Spanish social security register known as the Continuous Working Life Sample (CWLS). In addition to WLE, we also calculate the proportions of lifetime spent in unemployment, in inactivity or out of the labor market, and in retirement.

Our study contributes to the literature in several ways. First, to the best of our knowledge, we are the first to report working life table estimates for Spain. Second, we analyze differences by occupational category (manual vs. non-manual and skilled vs. unskilled), whereas the existing literature has tended to focus on education (for an exception see Leinonen et al. 2016). Both educational attainment and occupational categories affect WLE and complement each other, while also capturing different aspects of working life (Geyer et al. 2006). Third, we decompose WLE into contributions by age groups. For instance, let us assume that WLE is 35 years, i.e., that the average individual spends 35 years of her lifetime in employment. The contribution of the age group 20–29 could be 8 years. This means that eight of the 35 years of WLE are realized when individuals are in this age group, and the remaining 27 years are realized in other age groups. This type of calculation allows us to determine in which age groups years spent working are gained and lost over time, and thus, which groups should be targeted by policies.

Moreover, we assess the use of Sullivan’s method for the estimation of active working life expectancy (AWLE), capturing the lifetime spent in both employment and unemployment. To assess whether Sullivan’s method is appropriate for investigating working life, we compare the results of this approach with the findings from the Markov chain approach.

The remainder of this paper is structured as follows. An overview of the literature on WLE and a description of the Spanish context are given in Sect. 2. The dataset we use is described in Sect. 3. Our methods are introduced in Sect. 4. The results are presented in Sect. 5 and are discussed in Sect. 6. Section 7 concludes.

## Background

### Working Life Expectancy: Literature Review

Understanding work-life trajectories and how they relate to socio-demographic characteristics is an important task for aging societies with shrinking workforces. Surprisingly, much of the existing research on this topic has focused more on specific phases and transitions in the life course and less on complete work-life trajectories (Dudel and Myrskylä 2016).

One indicator that takes all of working life into account is working life expectancy (WLE). WLE is often defined as the expected proportion of lifetime spent in employment (e.g., Nurminen et al. 2005). Slightly different definitions of or names for this indicator are sometimes used, such as work-life expectancy or labor market life expectancy (Loichinger and Weber 2016); but the advantages of using such an indicator are the same: whereas other measures capture only parts of working life trajectories, WLE gives a complete picture of working life, covering all phases and labor market transitions during the life course. If, for instance, entry into the labor market is delayed, WLE will decrease. If, on the other hand, retirement is delayed, WLE will increase. If both the entry and the exit are delayed, WLE will capture the net effects of these delays and can account for all potential pathways in and out of work. Thus, WLE is a useful indicator of the sustainability of social security systems. Moreover, comparing WLE across socioeconomic groups provides us with insights into labor market inequalities and into how labor market (dis)advantages accumulate over the life course (Hayward and Lichter 1998). Related measures, such as the expected proportion of lifetime spent in retirement or the expected share of lifetime spent in unemployment, can be used to complement WLE and to broaden the scope of the analysis.

Most of the existing studies on length of working life have focused on the USA (e.g., Dudel and Myrskylä 2016; Skoog and Ciecka 2010; Millimet et al. 2010), although studies on WLE have also been conducted for Finland (Leinonen et al. 2016; Nurminen et al. 2005), the UK (Haberman and Bloomfield 1990; Butt et al. 2008), the Netherlands (Liefbroer and Henkens 1999), and Denmark (Hoem 1977). For instance, in their study for Finland, Nurminen et al. (2005) estimated that in 2006, WLE at age 16 was 29.7 years for females and 31.4 years for males; in other words, they found that 16-year-old Finnish females (males) could expect to spend 29.7 years (31.4 years) in employment. Leinonen et al. (2016) estimated that in 2012, WLE at age 50 was 9.1 years for Finnish males and 10 years for Finnish females. Most estimates for the USA have been higher. For example, Dudel and Myrskylä (2016) estimated that in 2008–2011 in the USA, WLE at age 50 was 12.7 years for males and 11.0 years for females, with strong educational gradients (see also Skoog and Ciecka 2010; Millimet et al. 2003). Strong educational gradients have also been reported for European countries (Leinonen et al. 2016).

The connection between WLE and economic conditions is a topic that has received little attention in the literature, as most studies focus on a single period, and do not assess changes in WLE over time. Dudel and Myrskylä (2016) studied the remaining WLE of US workers at age 50 over the 1992–2011 period. They found that the trend in remaining WLE at age 50 rose and fell depending on the general economic conditions of the period they studied. Their results showed, for example, that WLE declined during the recession of the early 2000s and during the Great Recession of 2007–2009.

### The Spanish Context

The Spanish labor market has been characterized as an insider-outsider labor market with high levels of unemployment in general and of youth unemployment in particular (Radl and Bernardi 2011). Nevertheless, employment was increasing rapidly from the mid-1990s until the crisis hit (Bernardi and Garrido 2006). As employment levels rose, the structure of the labor market changed: educational attainment has been rising with each successive cohort (Bernardi and Garrido 2006); and although the majority of Spanish workers are still employed in manual labor, the occupational structure has been shifting toward non-manual, qualified jobs (Oesch and Rodriguez Menes 2011).

After the recession hit in 2008, the unemployment rate in Spain increased sharply (according to Eurostat, $24.8\%$ in 2012) and remains high (19.6% in 2016). This trend was even more pronounced for young adults (Dolado et al. 2013). Several factors that likely contributed to the increase in unemployment in general and to the increase in youth unemployment in particular have been identified. These factors include the two-tier structure of the Spanish labor market, whereby some workers have highly protected employment contracts, while other workers have weakly protected fixed-term contracts (Bentolila et al. 2012a, b); and the high levels of skills mismatch and over-qualification, which are partly due to low levels of regional mobility (Dolado et al. 2013).

Moreover, much of the economic activity before the recession was in construction, an industry that employs large numbers of manual and low-skilled workers, and that declined sharply following the crisis (Jimeno and Santos 2014). This is one of the reasons why the impact of the crisis was heterogeneous, with some groups being less affected than others. For instance, older women and the better educated were less affected, while individuals who had low qualifications or were working in construction or manufacturing were hit hard (Chuliá et al. 2016).

In response to the crisis, several reforms of the social security system have been enacted (for an overview see OECD 2017), and substantial changes have been made to the pension system rules (Chuliá et al. 2016). A 2011 reform changed three of the retirement system’s main parameters: the number of years of earnings used to compute the retirement pension was expanded from the last 15 years to the last 25 years before retirement; the early retirement age was raised from 61 to 63; and, since 2013, the normal retirement age has been increasing annually and will continue to increase until it reaches 67 in 2027. Another reform enacted in 2013 raised the early retirement age further and introduced a sustainability factor that links pension replacement rates with life expectancy. In the past, disability pensions were often used as an alternative to early retirement, as they were less restrictive and more generous. However, recent reforms have reduced the attractiveness of this pathway out of the labor market (García-Gómez et al. 2012).

The Spanish labor market has also undergone considerable liberalization. The labor protections associated with permanent contracts were reduced in 2010 and again in 2012, while flexibility at the point of labor market entry was increased (Picot and Tassinari 2014). The system of unemployment benefits was subject to only minor and often temporary reforms until 2013 (Picot and Tassinari 2014). Levels of unemployment benefit coverage were comparatively low even before the crisis. Thus, many of the long-term unemployed lacked benefits (Venn 2012; Esser et al. 2013). However, a reform enacted in 2014 increased levels of income support and provided training for the long-term unemployed (OECD 2017).

When we combine the findings from the literature on WLE and the literature on the Spanish labor market and welfare state, we can make several predictions about how WLE developed in Spain over the study period. First, considering the intensity of the recession that hit Spain in 2008, we can expect to find that WLE declined considerably due to increased unemployment. Moreover, as unemployment is still high, we can anticipate that WLE had not returned to pre-recession levels by the end of the study period. Second, we can assume that the impact of the crisis was heterogeneous and differed across economic sectors and occupations. The findings of the existing literature imply that WLE changed more among manual and unskilled workers than among non-manual and skilled workers. Third, we can assume that a considerable share of WLE was lost at younger ages, as young adults were hit especially hard by the crisis, whereas older individuals with permanent contracts were less affected. Fourth, while most of the reforms discussed above may be expected to affect WLE, this influence is unlikely to be reflected in our results because the last year we cover in our analysis is 2013, which is around the time most of these reforms were enacted or went into effect.

## Data

The Continuous Working Life Sample (CWLS; Muestra Continua de Vida Laboral) is a $4\%$ random sample of the social security register in Spain, covering individuals who pay contributions or receive social security benefits (López Gómez et al. 2016). Data for contributing individuals are reported to the social security administration by employers and verified by the administration. The sample, which was extracted for the first time in 2004, contains labor trajectories dating as far back as 1981, and up through 2013. To ensure that individual follow-ups are possible, individuals remain in the sample from 2004 if they continue to be in contact with the social security system. Individuals lost due to death or administrative inactivity are replaced randomly until the sample reaches $4\%$. For individuals who are added to the sample as replacements, retrospective information is also available. Individuals lost in a certain year will enter the sample again if they become administratively active in subsequent years.

The dataset contains extensive information on each individual’s working life, including on his or her employment spells and spells of receiving social security benefits, occupational category, and date of death. Using this information, we assign each individual one of four labor force states for each year: employed, unemployed (receiving unemployment benefits), retired (receiving pension benefits), or inactive. Inactive means that the individual was under age 65 and not in contact with the social security system (e.g., that he or she was in education or a homemaker), or that the individual was receiving disability benefits. We assigned each individual’s labor force state based on the state in which he or she spent the most time during a year. For example, if during a given year an individual was unemployed for 7 months, employed for 4 months, and inactive for 1 month, the individual would be assigned the state “unemployed”. While it would in principle be possible to use monthly labor force states instead of annual labor force states, the adjustment procedures we apply are based on annual data and not compatible with monthly transitions (see Sect. 4.2). Inactive and unemployed individuals aged 65 and older are counted as retired, as individuals of retirement age are not entitled to unemployment benefits (Rebollo-Sanz 2012). Since people over age 80 are almost never in employment, individuals in this age group are always counted as retired.

The occupational category is divided into four categories according to the qualifications required for the job: skilled manual, skilled non-manual, unskilled manual, and unskilled non-manual. These four categories each combine several of eleven tax groups, which are reported by the employer. Although this variable is available for salaried workers only and not for self-employed workers or retirees, this lack of information is unproblematic for our analyses, as we focus on the highest occupational status ever obtained, which can be determined for most individuals in the sample (see below and Sect. 4.2). The “highest” occupational category is determined by assuming that non-manual is higher than manual and that skilled is higher than unskilled. For example, assume that an individual was employed in an unskilled non-manual position from 1995 to 2005 and in a skilled non-manual position thereafter. The highest occupational status ever attained by this person is skilled non-manual and does not change over time.

We use CWLS data from 2004 to 2013 for our analyses. Thus, we do not use the retrospective data before 2004. We made this choice because the data are selective regarding survival. For example, individuals who were retired during the 1980s and who died at the beginning of the 1990s are not covered by the 2004 sample. From 2004 to 2013, the CWLS contains information on 1,405,395 individuals. For our analyses, we use information on the 1,272,695 individuals—or roughly $91\%$ of the sample—to whom we could assign an occupational category. We employ a longitudinal perspective, focusing on the transitions of individuals between labor force states. In total, we observe 11,000,363 yearly transitions for the period 2004–2013.

## Methods

### Markov Chain Methods

To model the transition between labor force states (and death), we use a Markov chain approach (Hoem 1977; Millimet et al. 2003). More specifically, we specify a discrete-time Markov chain to estimate WLE and related measures. Let $Z_{t}$ denote the labor force state at time *t*. Markov chains are based on transition probabilities $\mathrm {Pr}(Z_{t+1}=i|Z_{t}=j)=p_{ij}$, which capture the probability of being in state *i* at time $t+1$ conditional on being in state *j* at time *t*. All results are derived from these transition probabilities. As we noted above, we distinguish between four labor force states: employed, unemployed, inactive, and retired. Individuals move between these states according to the transition probabilities. In addition to the state at time *t*, we assume that transition probabilities differ by gender, age, calendar year, and occupational category. We assume that 99 years is the highest age that can be reached and that all individuals aged 99 will die.

Based on Markov chains, we estimate the life expectancy in each of the four labor force states (e.g., Kemeny and Snell 1971): WLE, life expectancy in unemployment, life expectancy in retirement, and inactive life expectancy. If not stated otherwise, our results are life expectancies at age 15, meaning that the life expectancies in each of the four labor force states sum to the total life expectancy at age 15. In addition, we decompose WLE by six age groups (19 or younger, 20–29, 30–39, 40–49, 50–59, 60 or older), giving the contribution of each age group to total WLE.

### Estimation and Adjustment of Transition Probabilities

To estimate transition probabilities $\mathrm {Pr}(Z_{t+1}=i|Z_{t}=j)$, we use multinomial logistic regression (Greene 2012). The state at time $t+1$ is used as the dependent variable, and the state at time *t* is used as one of the explanatory variables. Age is included as a cubic smoothing spline, which does not restrict the effect of age to a specific functional form (Yee and Wild 1996). The highest occupational category ever attained is included via dummy variables. We use data of the years 2004–2013 and control for trends by including dummy variables for years using 2004 as a reference. We analyze transitions from *t* to $t+1$. This means, for example, that the dummy for 2006 relates to the transition from 2006 to 2007. Thus, no dummy is included for 2013, as it is only used as year $t+1$ in conjunction with data from 2012. We also use interaction terms of occupational category and year (for interactions with age and gender, see below).

The regression model outlined above is applied to each of 10 subsamples, which are generated by splitting the data by gender and by the following age groups: 15–29, 30–54, 55–64, 65–79, and 80–99. The first age group corresponds to the transition from education to the labor market and early working life. Age 30–54 covers the larger part of working life, while the transition to retirement occurs in the age range of 55–64. Age 65–79 corresponds to retirement and age 80–99 to advanced age. Among the advantages of splitting the dataset and applying regression separately to each subsample are that doing so allows for discontinuities in the otherwise smooth age schedule and for the introduction of implicit interactions of age and gender with all explanatory variables. It also reduces the computational burden. As the size of the CWLS is large, using this approach does not lead to a notable loss of efficiency. Individual subsamples are still sizable, and the smallest subsample consists of roughly 59,000 transitions, while the largest has 3,100,000 transitions.

As was already noted in Sect. 3, we are able to assign the highest occupational category ever attained to roughly $91\%$ of the individuals covered by the CWLS. To assess whether missing information on occupational category affects our findings, we conducted sensitivity analyses which show that missingness is likely to be non-selective and can be safely ignored. A description and results of the sensitivity analysis are given in the supplementary materials.

The dataset has two other potential issues. First, the dataset only covers individuals who are in contact with the social security system. Second, it is not possible to distinguish between moving from one of the “social security states” to inactivity or outmigration. To deal with these issues, we adjusted transition probabilities using additional data taken from the (HMD 2015) and from the Instituto Nacional de Estadística. Among other things, the adjustment procedure matches our results for life expectancy with HMD life tables. For details of the methodology and additional results and sensitivity tests, see the supplementary materials.

### Sullivan’s Method

In contrast to the Markov chain approach, Sullivan’s method is based not on transition probabilities, but on prevalence rates. Formally, the remaining life expectancy spent in a state *j* at age *x* can be calculated as

$$\begin{aligned} e_j(x)=l_x\sum \limits _{k=x}^{99} L_k d_j(k), \end{aligned}$$

where $l_x$ is used to denote the survivor function of a life table, $L_x$ gives the person-years lived at age *x* derived from the same life table, and $d_j(x)$ gives the proportion of individuals in state *j* at age *x* (Sullivan 1971). Essentially, future expected lifetime is weighted by the probability of being in state *j*.

A detailed discussion of the assumptions needed to apply Sullivan’s method is presented in Imai and Soneji (2007). The results obtained using Sullivan’s method may differ from the results obtained using Markov chains, as the former are based on prevalence rates, $d_j$, while the latter are based on transition probabilities, $p_{ij}$, and the prevalence rates might not fully reflect the transition probabilities (Mathers and Robine 1997).

As an example, assume we are interested in the time spent in employment in a strongly segregated labor market in which employed individuals have a high probability of staying employed, while unemployed individuals mostly stay unemployed. For people entering the labor market, there are two possible tracks: one of employment and one of unemployment. Now assume that between two periods the probability of entering the employment track decreases drastically for young individuals because of adverse economic conditions. In this scenario, employment prevalence will decrease for young individuals only, and Sullivan’s method will thus show no dramatic change, whereas the Markov chain approach will capture the shift toward more individuals being on the unemployment track and will thus show a strong drop in employment expectancy.

To compare Sullivan’s method with the Markov chain approach, we directly compare our findings to estimates from Eurostat, which are based on Eurostat life tables and age-specific labor force participation rates derived from the EU labor force survey (for details see Eurostat 2016). As the labor force participation rates of the EU labor force survey capture both time spent in employment and time spent in unemployment, and thus AWLE, we combine our estimates of life expectancy spent in employment and unemployment to calculate life expectancy spent in the labor force. Labor force participation rates also count as part of the labor force individuals who are not receiving unemployment benefits but are looking for employment, whereas the CWLS does not include these individuals. Thus, these individuals are counted as inactive in our analysis. As this use of different definitions could lead to differences in the results, we also calculated labor force participation rates using the CWLS and combined these rates with life tables for Spain obtained from the Human Mortality Database (2015) to calculating AWLE using Sullivan’s method.

All calculations were conducted using R (R Core Team 2015). Moreover, the VGAM package for R was used (Yee 2010). Figures were created using ggplot2 (Wickham 2009).

## Results

### Transitions and Transition Probabilities

Table 1 gives an overview of the number of individuals and the number of transitions by gender and occupational category. As males have a higher labor force attachment than females, there are more males than females in the CWLS. The structure of the sample with regard to occupational category is skewed in favor of unskilled and manual work. This reflects the structure of the Spanish labor market, which is dominated by labor-intensive industries with a high demand for unskilled work (construction, tourism, personal services; see Bentolila et al. 2012b).

**Table 1:** Number of individuals and transitions by gender and occupational category. *Source*: Own calculation, CWLS 2004–2013

	Individuals	Transitions	Relative (%)
By gender			
Male	722,333	6,189,028	57
Female	550,362	4,811,335	43
By occupational category			
Skilled non-manual	74,957	654,032	6
Skilled manual	144,817	1,222,560	11
Unskilled non-manual	305,075	2,650,622	24
Unskilled manual	747,846	6,473,149	59
Total	1,272,695	11,000,363	100

**Fig. 1:**
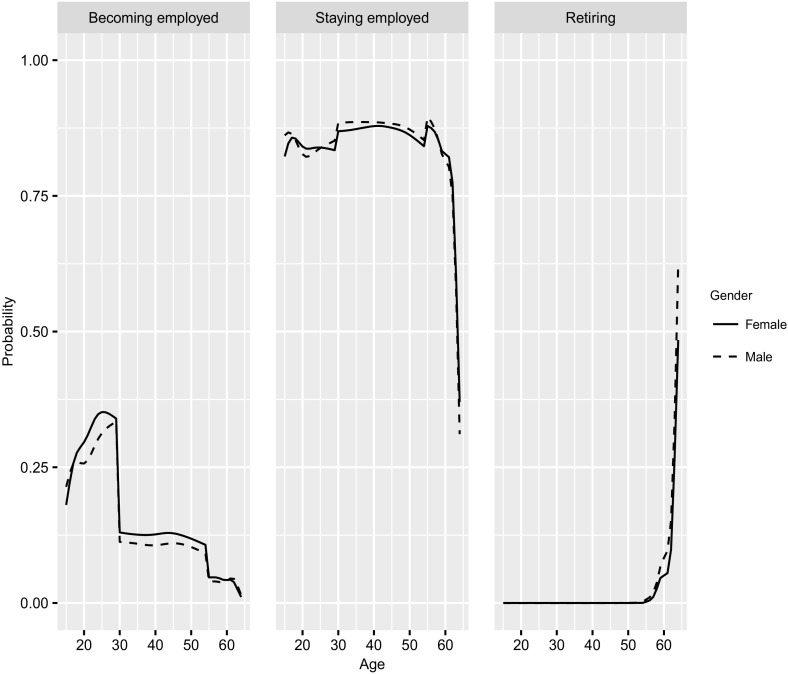
Transition probabilities by age (15–64) and gender: probability of becoming employed conditional on being inactive; probability of staying employed; probability of retiring out of employment; 2012/2013. *Source*: Own calculation, CWLS 2004–2013

Figure 1 shows the transition probabilities for key transitions based on data of the years 2012 and 2013. The first panel shows the probability of becoming employed conditional on being inactive by age and gender, whereby only findings for the main working ages of 15–64 are shown. The second panel shows the probability of staying employed conditional on being employed, and the third panel shows the probability of retiring conditional on being employed, i.e., of leaving the labor market.

The probability of becoming employed conditional on being inactive increases relatively steadily starting at age 15 and peaks at age 25 for females and at age 29 for males. For example, the probability of becoming employed at age 29 reflects the transition from being inactive at age 29 in 2012 to being employed at age 30 in 2013. Females have a higher probability of becoming employed than males for most ages up to age 29, except for ages 15 and 16. This difference reflects the fact that females spend more time in education and enter the labor market later than males (Dolado et al. 2000). The lagged peak and lower level among males may arise if significant numbers of inactive males are unemployed but are not receiving unemployment benefits, as the eligibility criteria are strict and a minimum amount of contributions is needed to qualify (Venn 2012). A strong break occurs for both males and females at age 30. This discontinuity is due to the estimation of transition probabilities by subsamples. Estimating transition probabilities using only one sample instead of multiple subsamples could lead to potentially biased estimates, as this would restrict results to a strictly smooth schedule, whereas our approach loosens this smoothness requirement and allows for breaks. While the resulting piecewise smooth schedule overall is less restrictive, it might slightly exaggerate discontinuities in the schedule. Up to age 54 the transition probabilities do not change much and then decrease rapidly.

The probability of staying employed is lower for individuals up to age 30 ($85\%$) than for individuals up to age 54 (almost $90\%$). This gap is attributable to the fact that younger individuals are more likely than older workers to be on a fixed-term contract. This pattern might also explain the higher probability of staying employed found for males than for females (Azmat et al. 2006). Starting at age 60, the probability of staying employed decreases rapidly due to retirement. This trend is mirrored in the probability of retiring, which increases sharply starting at age 60.

### Results on Working Life Expectancy for the Total Population

Before we break down WLE by occupational category, we present findings for the total male and the total female population, which give an overview of the general trends in Spain. These results are based on estimates of transition probabilities that do not include occupational category as an explanatory variable. Findings are shown in Table 2. Note that due to our methodological approach, each row of the table relates to 2 years *t* and $t+1$; e.g., 2004 and 2005. As the recession hit Spain in 2008, 2007/2008 can be seen as a “mixed” period, which arises from using year-to-year transitions, while the years before this point can be viewed as a pre-crisis period, and the years after this point can be seen as a period marked by recession.

**Table 2:** Remaining life expectancy at age 15 (in years) spent in employment, unemployment, inactivity, and retirement, for Spanish males and females by period. *Source*: Own calculation, CWLS 2004–2013

	Employed	Unemployed	Inactive	Retired	Total
Males					
2004/2005	37.8	2.4	5.8	16.5	62.5
2005/2006	36.5	2.2	7.7	16.1	62.2
2006/2007	37.5	2.6	6.5	16.6	63.1
2007/2008	32.1	4.0	10.8	16.4	63.0
2008/2009	25.6	6.7	14.6	16.7	63.6
2009/2010	28.5	6.4	12.2	17.0	64.0
2010/2011	29.3	5.6	12.3	17.3	64.4
2011/2012	26.1	6.2	15.1	17.2	64.5
2012/2013	28.8	5.9	12.6	17.4	64.6
Females					
2004/2005	32.8	3.0	12.3	20.9	68.5
2005/2006	30.5	2.7	15.3	20.5	69.0
2006/2007	32.6	3.6	12.3	21.0	69.3
2007/2008	30.0	4.1	14.6	20.8	69.5
2008/2009	25.6	5.4	18.1	20.5	69.6
2009/2010	26.6	5.9	16.6	20.9	69.6
2010/2011	27.2	5.3	16.8	21.1	69.7
2011/2012	24.9	5.4	18.7	21.3	70.3
2012/2013	24.7	4.8	19.6	21.3	70.3

All of these numbers can be interpreted similar to total life expectancy and represent the average time spent in each state (measured in years) by members of a hypothetical cohort who experience the transition probabilities of a specific period. Because of the method we apply to correct mortality, the total life expectancy is similar to the life table estimates from the HMD, albeit with marginal differences: e.g., according to our findings, remaining life expectancy at age 15 for females in 2012/2013 is 70.33 years, while the corresponding value reported by the HMD for 2012 is 70.41.

Because of the recession, WLE has decreased considerably for both males and females. WLE was roughly 38 years for males and 33 years for females in 2006/2007, but had declined to 26 years for both males and females in 2008/2009. This implies that males lost nearly 12 years of WLE while females lost 7 years due to the recession. WLE varied before and during the crisis, but these year-by-year differences are small compared to the impact of the recession.

The years of WLE lost due to the recession are mostly spent in either unemployment or inactivity. Unlike the time spent in unemployment and inactivity, the time spent in retirement has not been greatly affected by the recession for either males or females, and recent reforms do not show clear effects, at least yet. Women spent four more years in retirement than men, mostly due to their longer life expectancy.

### Working Life Expectancy by Occupational Category

**Fig. 2:**
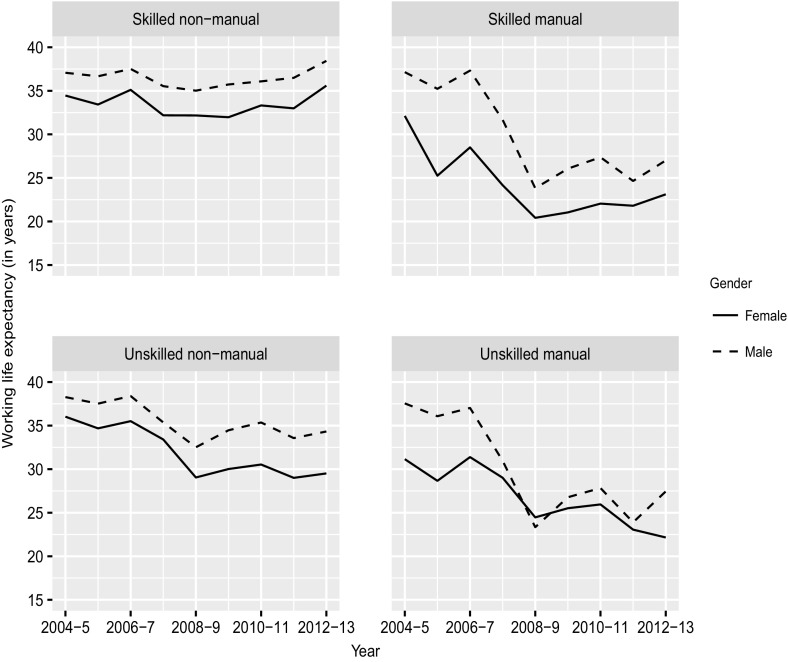
Remaining life expectancy in employment (WLE) at age 15, 2004 to 2012 by occupational category and gender. *Source*: Own calculation, CWLS 2004–2013

Estimates of WLE (measured in years) by occupational category and gender are shown in Fig. 2. These results only include years spent in employment. Additional tables showing life expectancy in all labor force states by gender and occupational category are provided in the supplementary materials.

For males before the recession, both unskilled manual and non-manual workers had a WLE of roughly 38 years (2004/2005 value), while both skilled non-manual and manual individuals had a slightly lower WLE of 37 years. The impact of the Great Recession differed considerably by occupational category and reversed the gap between skilled and unskilled male workers. The changes in WLE between 2006/2007 and 2008/2009 amounted to 2.5 years for skilled non-manual work, 5.9 years for unskilled non-manual work, and close to 14 years for both skilled and unskilled manual work. For the most recent period (2012/2013), WLE is still below pre-recession levels for skilled manual, unskilled manual, and unskilled non-manual male workers, with values of 27 years, 27 years, and 34 years, respectively. For skilled non-manual workers, WLE is above the pre-recession level, with a value of 38 years.

The results for females roughly follow those of males, but mostly at a lower level. For instance, in 2004/2005 WLE was 37 years for male and 32 years for female skilled manual workers. Interestingly, the gender gap in WLE decreased considerably for manual work, while it increased slightly for non-manual work. The gender gap is calculated as the WLE of males minus the WLE of females. For example, the gender gap for unskilled manual workers was 5.6 years in 2006/2007 and 1.1 years in 2008/2009. For skilled manual workers, these values were 8.8 and 3.4, respectively. The recession thus had a greater impact on females than on males working in non-manual jobs, but a smaller impact on females than on males working in manual jobs.

### Decomposition of Working Life Expectancy by Age Group

**Fig. 3:**
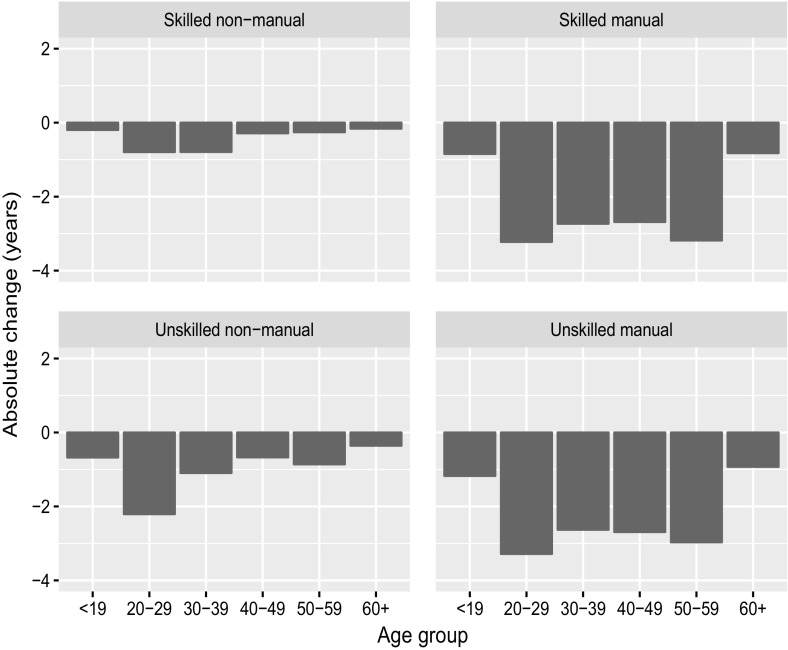
Decomposition of change in WLE between 2006/2007 and 2008/2009 by age group for males by occupational category. *Source*: Own calculation, CWLS 2004–2013

**Fig. 4:**
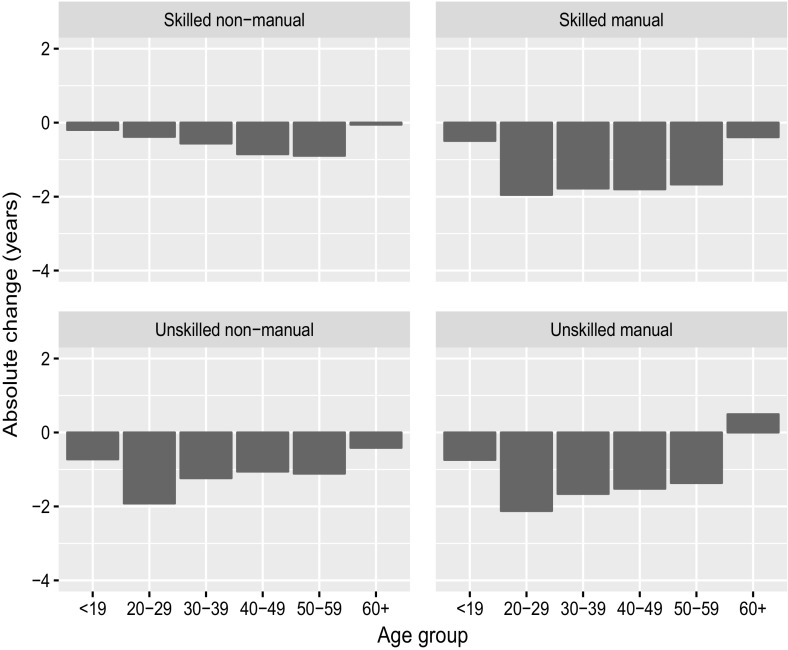
Decomposition of change in WLE between 2006/2007 and 2008/2009 by age group for females by occupational category. *Source*: Own calculation, CWLS 2004–2013

The WLE losses may be concentrated in specific age groups. For instance, we can speculate that because of high youth unemployment, most of the years lost are concentrated at younger ages. Figures 3 and 4 present a decomposition of the changes in WLE between 2006/2007 and 2008/2009 by age class for males and females, and for all occupational categories. The bars in each plot indicate how the differences in WLE are distributed among the following age classes: 19 or younger, 20–29, 30–39, 40–49, 50–59, and 60 or older. For instance, the total WLE of skilled manual male workers decreased by 13.5 years, and 2.7 of these years were lost in the age group 30–39. Detailed results are provided in the supplementary materials.

For neither males nor females, the age groups 19 or younger and 60 or older contributed substantially to WLE, and the losses are small. The same holds by occupational category. If we focus on the prime working age groups, we can see that for male skilled and unskilled manual workers, the losses are relatively evenly distributed among the age classes, although the losses are slightly larger in the age groups 20–29 and 50–59. For male skilled and unskilled non-manual workers, the losses are more concentrated in the younger age groups. Thus, for males the impact of youth unemployment on WLE seems to be limited to non-manual workers. Interestingly, for females the losses are more concentrated in the younger age groups for unskilled but not for skilled workers. For skilled non-manual female workers, the losses even show the opposite pattern and are concentrated at higher ages. The total loss of WLE is, however, small for this group.

### Active Working Life Expectancy and Sullivan’s Method

**Fig. 5:**
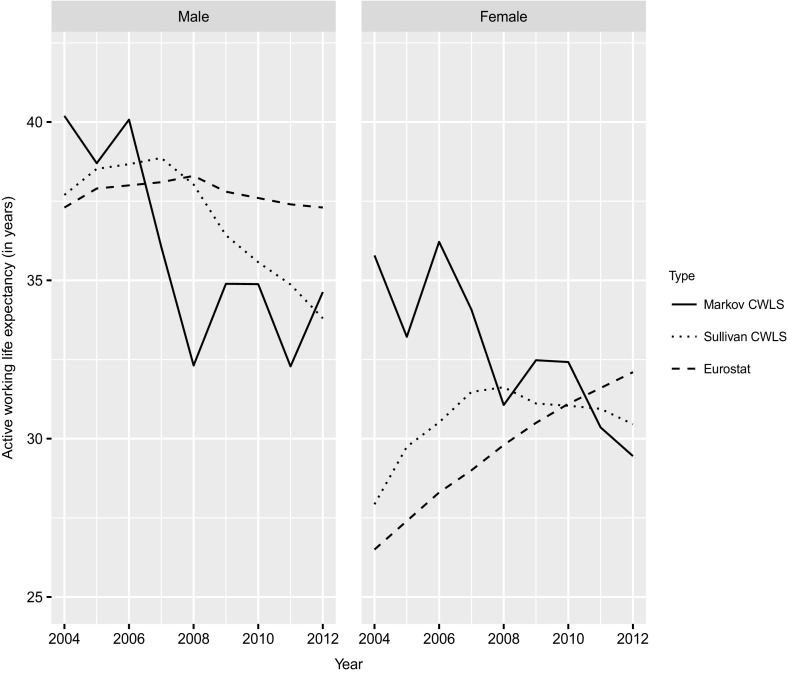
Average time spent in the labor force (AWLE; in years) 2004–2012 by year and gender, calculated using transition probabilities and Markov chains (Markov CWLS); participation rates obtained from the CWLS and Sullivan’s method (Sullivan CWLS); and estimates provided by Eurostat based on Sullivan’s method. *Source*: Eurostat; own calculation, CWLS 2004–2013

Figure 5 compares the estimates of active working life expectancy (AWLE) based on Markov chains with the estimates obtained using Sullivan’s method; i.e., the Eurostat estimates and the estimates based on the CWLS, as described in Sect. 4.3. The Markov chain estimates are calculated by adding the columns “Employed” and “Unemployed” of Table 2. Note that for the estimates obtained from Markov chains, each data point in the figure relates to 2 years, but the axis labels only show the first year. For instance, the data point for 2012 actually relates to 2012 as *t* and to 2013 as $t+1$. For the results based on Sullivan’s method, each data point relates to 1 year as usual.

For males, the differences between our estimates based on Markov chains and the Eurostat estimates are rather large. The biggest difference is found for 2008/2009, with the Eurostat estimates being nearly 6 years higher than our estimates. While our findings show a decline in male AWLE of roughly 12 years between 2006/2007 and 2008/2009, the Eurostat estimates actually show an increase of 0.3 years between 2006 and 2008, and the values are generally rather stable. As we discussed above, this discrepancy may be at least partly due to the fact that the Eurostat estimates are based on labor force participation rates, which also include individuals who are not receiving unemployment benefits but are looking for work, whereas the CWLS only allows us to cover individuals who are receiving unemployment benefits. The labor force participation rates calculated from the CWLS thus differ from the Eurostat estimates. AWLE calculated using CWLS data shows a decline for males, but the decrease is later and slower than in the Markov estimates; i.e., a decline of just 0.6 years between 2006 and 2008. Thus, this estimate differs from the estimate generated using the Markov chain approach, again by roughly 6 years.

For females, the results also differ strongly depending on whether the Markov approach or Sullivan’s method is used. Both our estimates and Eurostat’s estimates obtained using Sullivan’s method show an increase in WLE, at least from 2004 to 2008, while the Markov chain approach shows a decrease. After 2008, all of the estimates are roughly at the same level for at least some years, but the trends still differ: the estimates obtained using the Markov chain approach show a decline of roughly 1.6 years between 2008/2009 and 2012/2013, the estimates obtained using Sullivan’s method based on CWLS data show a decline of 1.1 years between 2008 and 2012, and the Eurostat estimates show an increase of 2.3 years. Overall, Sullivan’s method seems to miss both the levels and the trends of AWLE.

## Discussion

### Main Findings

Our results show that the recession had a strong negative effect on working life expectancy (WLE) in Spain, and WLE did not recover to pre-recession levels until 2013. The large gender difference in the impact of the recession is at least partly attributable to differences in the distribution of the sexes across economic sectors, as more men than women work in sectors like construction that have been hit especially hard by the recession (Bentolila et al. 2012a). The lost lifetime in employment is spent not just in unemployment, but in inactivity. The increase in inactivity can be attributed to several factors. First, young Spaniards are staying in the educational system longer to increase their chances in the labor market and to avoid unemployment (Dolado et al. 2013). Second, some individuals may withdraw from the labor market at least temporarily if they are not entitled to unemployment benefits and do not expect to find a job (Congregado et al. 2011). Third, unemployed individuals may lose their eligibility for unemployment benefits after a certain period of time (Venn 2012) and are then counted as inactive. The lifetime spent in retirement did not change due to the recession. But major pension reforms were enacted or took effect in 2013, suggesting that changes might have occurred after the period we study.

If we break down WLE by occupational category, we see that the impact of the recession has differed considerably across these groups. This finding is consistent with earlier literature on the Spanish labor market (Radl and Bernardi 2011; Radl 2013), showing strong segregation by industry, occupation, and education, and the results of the emerging literature on the crisis in Spain, which has shown that sectors dominated by low-skilled work have been hit harder than others (Bentolila et al. 2012a). More surprising is the result that before the recession unskilled workers had a slightly higher WLE than skilled workers, as most research has shown that having a higher social class or level of education is associated with having a higher WLE (Leinonen et al. 2016; Millimet et al. 2010; for an exception see Liefbroer and Henkens 1999). As the highest occupational category ever attained is correlated with education—e.g., skilled non-manual workers will on average have a higher education than unskilled manual workers—we expected to find similar results for Spain. Our unanticipated finding may be explained by the interplay of two factors. First, because skilled individuals have to spend more time in the educational system than unskilled workers, they spend more time in the inactive state when they are young. Second, in 2004/2005 unemployment was extremely low by Spanish standards due to a boom in construction and market services that generated a large demand for unskilled work (Bentolila et al. 2012a; Jimeno and Santos 2014). Because of the latter factor, employment levels were extremely high for unskilled workers in the pre-recession period covered in our dataset.

When we decompose the effect of the recession by age groups, we find that for some occupational categories (skilled/unskilled non-manual males; unskilled manual/non-manual females) younger people have been harder hit than older people, as would be expected given the high rates of youth unemployment (Dolado et al. 2013). For skilled and unskilled manual male workers, the losses are distributed relatively evenly among age groups, although the effects are slightly stronger for the youngest and the oldest workers. This distribution may be attributable to the tremendous impact of the recession on sectors that employ large numbers of male manual workers, hitting young and old workers alike. The finding that skilled non-manual females lost the most WLE at higher ages is surprising, but their overall loss in WLE is small.

Compared to findings from the literature, our results indicate that WLE in Spain at age 15 was relatively high before the start of the recession and relatively low thereafter. For Finland for the year 2006, Nurminen et al. (2005) reported estimates of WLE at age 16 of 29.7 years for females and 31.4 years for males. Our estimates for 2006/2007 are 32.6 years for females and 37.5 years for males, while our estimates for 2012/2013 (the most recent period for which we have data) are 24.7 years for females and 28.8 years for males. This discrepancy may be attributable to the fact that before the recession unemployment was low in Spain and the demand for unskilled labor was high.

### Methodological Considerations

Our comparison of Sullivan’s method with Markov chains showed that Sullivan’s method might lead to potentially misleading estimates of both the trends and the levels of AWLE. The results obtained using Sullivan’s method show no or a lagged effect of the recession on males and an increase in AWLE for females, while the results obtained using Markov chains show a strong impact of the recession and a decrease in AWLE for both males and females. As results of Markov chains are generally considered to be a valid benchmark for Sullivan’s method (e.g., Mathers and Robine 1997), our findings show that Sullivan’s method might be inappropriate if the underlying transition probabilities are changing fast, as was the case for employment during the recession.

A potential question that may be raised about our results is whether we have been able to estimate the effect of the recession, as changes in WLE may be affected not only by the crisis, but by other factors as well. While we cannot rule out the effects of other factors, it is plausible that the drastic changes we find are at least partly attributable to the recession: the crisis can be seen as an exogenous shock; the change in WLE around the time of this exogenous shock was strong, whereas before and after the crisis hit annual variation was comparatively small. Moreover, there were no major labor market or social security reforms enacted shortly before the crisis, which could affect our estimates. Given that the period of 2007/2008 was only partly affected by the crisis—which fully hit the labor market only in 2008—comparing 2006/2007 and 2008/2009 to assess the impact of the crisis should at least yield a good approximation. Things are less clear for the effects of recent reforms, such as the pension reforms of 2011 and 2013. While we do not see clear-cut impacts of these reforms, their effects might cancel out with other factors, or they might take effect with delay, after the period we study.

Several caveats regarding our findings are in order. First, all our findings are based on a period perspective and show what would happen to a hypothetical cohort if the conditions of a specific period prevailed. While this approach can provide useful information on the conditions of a period, our results may not directly translate to the experiences of real birth cohorts (Leinonen et al. 2016). Second, not distinguishing between part- and full-time employment may be problematic, especially for women. Our findings show that the recession had a less severe impact on females, but this could partly be due to a reduction in full-time work and an increase in part-time work. Third, we combine disability with labor market inactivity (before age 65) or retirement (after age 65) to one state. Having separate states instead would be more informative, but this would increase the size of the state space and as a consequence increase the number of model parameters and transition probabilities considerably, potentially leading to sparsity issues. Fourth, while we distinguish between gender and occupational category, several other variables important for labor market outcomes were not accounted for, such as migration status (Mooi-Reci and Muñoz Comet 2016).

## Conclusions

Our findings show that the Great Recession has had a strong impact on working trajectories in Spain and that working life expectancy (WLE) declined rapidly. However, the effects of the recession on WLE vary considerably by occupational category and gender. Skilled non-manual workers have been only slightly affected, while unskilled manual workers have lost a very large number of years of WLE. Women have been less affected by the recession than men. Decomposing losses in WLE by age groups showed that the WLE lost at younger ages strongly depends on the occupational category and that—at least for some categories—it is roughly comparable to or only slightly higher than the WLE lost at older ages. Using Sullivan’s method to calculate life expectancy spent in the labor force leads to estimates that are quite different in terms of both levels and trends and might be misleading.

While younger and middle-aged individuals may be able to catch up on lifetime worked if economic conditions improve, there are still potential risks like scarring effects; a term that refers to the negative effects of unemployment spells on later labor market performance (Bell and Blanchflower 2011). Older individuals who experience reduced time spent in employment may have insufficient opportunities to catch up before retirement. In light of population aging, this loss of WLE seems rather troublesome, as there is already substantial pressure on the Spanish social security system in general and its pension system in particular (Sánchez Martín and Sánchez Marcos 2010; Díaz-Giménenz and Díaz-Saavedra 2009). Given our findings, it remains to be seen whether recent reforms—e.g., the increase in the retirement age from 65 to 67 over the period of 2013–2027, or additional support for long-term unemployed—will lead to a notable increase in the length of working life in the future which will be able to counter the reduction in WLE due to the recession.

Potential avenues for future study include investigating the broader question of how economic conditions in general and the financial crisis in particular affect WLE. While our findings indeed show that the recession has had a strong impact, it remains to be seen if this will also be true from a cohort perspective and for other countries and institutional settings.

## Electronic supplementary material

Below is the link to the electronic supplementary material.

Supplementary material 1 (pdf 207 KB)
