# Supplementary material for: The Length of Working Life in Spain: Levels, Recent Trends, and the Impact of the Financial Crisis
Source: Eur J Popul. 2018 Jan 3;34(5):769–91. doi: 10.1007/s10680-017-9458-9 (PMC6261850; doi:10.1007/s10680-017-9458-9)
Supplement: Supplementary file 1 — Supplementary material 1 (pdf 207 KB) [file 10680_2017_9458_MOESM1_ESM.pdf]

The Length of Working Life in Spain:
Levels, Recent Trends, and the Impact of the Financial Crisis
-Online supplementary materials-

These supplementary materials include methodological details and additional findings. Section A describes the adjustment procedures mentioned in section 4.2 of the main paper in detail. Section B includes results of the sensitivity analysis also mentioned in section 4.2 of the main paper. Section C gives more detailed findings than section 5 of the main paper.

A. Correcting transition probabilities

A.1. Correcting for selectivity regarding inactivity

A potential issue of the Continuous Working Life Sample (CWLS) is selectivity, as it only covers individuals who are in contact with the social security system. Specifically, the transition probabilities for which the labor force state equals inactive might be biased. For example, the probability of transitioning from inactivity to employment might be biased upwards, as individuals who stay inactive for the whole period of 2004 to 2013 are not included in our dataset. Note, however, that valid estimates can be attained for the transition probabilities conditional on being employed, unemployed, or retired.

To test whether selectivity of the dataset affects the results, we combined the CWLS with data on the total population by age and gender taken from the Human Mortality Database (HMD; 2015), and estimated the transition probabilities using a nonparametric approach. Our basic reasoning for performing this test is that the nonparametric approach should give reliable estimates, and is thus suitable for use as a benchmark to which we can compare our findings. Note that the nonparametric approach cannot be used for calculations by occupational category, as the HMD includes information by age and gender only, and not by occupational category. We therefore compared the results for the total population. Moreover, we also applied the approach of Dudel and Myrskylä (2016) to the survival probabilities.

Adjustment of transition probabilities for the inactive state to correct for selectivity of the CWLS proceeds in the following fashion. First, transition probabilities are estimated based on CWLS data and population data taken from the HMD. More specifically, for each year the CWLS is used to estimate the size and structure of the population by age, gender, and in the labor force states employed, unemployed, retired, and disabled. This is done by weighting every observation available for a specific year by its inverse selection probability $100/4$. Let $n_{t,a,g,s}$ denote the population counts derived this way where t indicates the year, a the age $(0,\ldots,99)$, g the gender, and s the labor force state. Let $n_{t,a,g}$ be the total population for age a, gender g, and year t obtained from the HMD. The number of inactive individuals can be calculated as

$$n_{t,a,g,\texttt{inactive}} = n_{t,a,g} - \left(n_{t,a,g,\texttt{employed}} + n_{t,a,g,\texttt{unemployed}} + n_{t,a,g,\texttt{retired}} + n_{t,a,g,\texttt{disabled}}\right).$$

Transition probabilities are calculated as

$$p_{ij} = \frac{n_{t,a,g,j}^{t+1,a+1,g,i}}{n_{t,a,g,j}},$$

where $n_{t,a,g,j}^{t+1,a+1,g,i}$ is the number of individuals who are in state j and age a at time t and in state j and age $a+1$ at time $t+1$. To calculate these for j being equal to inactive first transitions to the labor force states employed, unemployed, retired, and disabled are calculated. This is done by counting observations in the CWLS who are in state i at $t+1$ and were inactive at time t and weighting these observations with $100/4$. Then probabilities of dying are calculated for the inactive state, which requires $n_{t,a,g,j}^{t+1,\texttt{dead}}$. This number can be derived by exploiting the fact that

$$n_{t,a,g}^{t+1,\texttt{dead}} = n_{t,a,g,\texttt{employed}}^{t+1,\texttt{dead}} + n_{t,a,g,\texttt{unemployed}}^{t+1,\texttt{dead}} + n_{t,a,g,\texttt{retired}}^{t+1,\texttt{dead}} + n_{t,a,g,\texttt{disabled}}^{t+1,\texttt{dead}} + n_{t,a,g,\texttt{inactive}}^{t+1,\texttt{dead}}.$$

The first four quantities on the right hand side can be estimated using the CWLS, while $n_{t,a,g}^{t+1,\mathtt{dead}}$ is taken from the HMD. Finally, the probability of staying inactive is calculated by using the fact that $\sum_i p_{ij} = 1$.

In a second step, nonparametric estimates are used to adjust semiparametric estimates of transition probabilities starting in labor force state inactive by occupational category. Let $p_{j,a,t,g,o}$ be the probability of being in state inactive in age a at time t for gender g and occupational category o and moving to the state j (e.g. employed) in age $a+1$ at time $t+1$. Assume that this probability has been estimated using the semiparametric approach. Let $\mathrm{P}_{a,t,g}(o)$ be the proportion of inactive individuals in age a at time t and of gender g and of occupational category o, which is estimated using the CWLS. The probability of moving to state j unconditional on occupational category is given by

$$p_{j,a,t,g} = \sum_o p_{j,a,t,g,o} \mathrm{P}_{a,t,g}(o)$$

Let $p^*_{j,a,t,g}$ be the estimate of the unconditional probability obtained by applying the nonparametric approach outlined above. Semiparametric estimates $p_{j,a,t,g,o}$ are then multiplied by a correction factor $a_{j,a,t,g}$ which is calculated as

$$a_{j,a,t,g} = \frac{p^*_{j,a,t,g}}{p_{j,a,t,g}}.$$

Adjusted transition probabilities $p^A_{j,a,t,g,o}$ are then calculated as

$$p^A_{j,a,t,g,o} = p_{j,a,t,g,o} a_{j,a,t,g} c_{j,a,t,g,o},$$

where $c_{j,a,t,g,o} = 1/\left(\sum_j a_{j,a,t,g} p_{j,a,t,g,o}\right)$ is an additional scaling factor which guarantees that for each occupational category $\sum_j p^A_{j,a,t,g,o} = 1$ holds. Because of this additional scaling factor

$$p^*_{j,a,t,g} = \sum_o p^A_{j,a,t,g,o} \mathrm{P}_{a,t,g}(o)$$

will only hold approximately.

Note that this approach based on indirect nonparametric estimates is not perfect. Our reasoning is that the CWLS should give a relatively complete picture of working trajectories of males as they typically have high labor force participation rates and most of them will either be employed, unemployed, disabled, or retired, implying that the semiparametric estimates of transition probabilities based on the CWLS should lead to reasonable results, i.e. results which are only slightly biased, if at all. If estimates of WLE derived completely nonparametrically coincide with those of the semiparametric approach and adjustment does not change the results of the semiparametric approach much, then the nonparametric estimates should not be totally off. If this is the case for males, completely nonparametric estimates of WLE for females can serve as a benchmark for unadjusted semiparametric estimates to assess whether selectivity of the social security population is strong and they can be used to assess whether the adjustment method outlined above leads to reasonable results.

A.2. Correcting for out migration

Another potential issue of the CWLS is that it is not possible to distinguish between moving from one of the "social security states" to inactivity and outmigration. For instance, assume that an individual is employed and is thus in contact with the social security system in 2004, and then moves abroad. Another individual is employed in 2004 and becomes inactive thereafter. In both cases, there are no entries in the social security data after 2004. Simply assuming that both individuals are inactive will lead to a potentially large overestimation of the probability of moving to inactivity, as the levels of outmigration were sizable for at least some of the years of our study period (Larramona, 2013; Izquierdo et al, 2015).

To deal with this issue, we first calculated out migration probabilities by age and gender using population counts obtained from the HMD and out-migration counts obtained from the Estadística de Variaciones and the Estadística de Migraciones (see below). Let $m_{t,a,g}$ denote these out-migration probabilities. Let $p^A_{i,a,t,g,o}$ denote the adjusted probability of being in state i at time t and occupational category o and moving to the state inactive at $t+1$ (adjusted using the method described in A.1). These adjusted probabilities will then be corrected via $p^A_{i,a,t,g,o} - m_{t,a,g}$, yielding probabilities $p^M_{i,a,t,g,o}$. In a second step, transition probabilities are rescaled such that they sum to survival probabilities. These rescaled probabilities are then used for calculating WLE and so on.

While the data from the Estadística de Migraciones (EM) is considered to be better than the data from the Estadística de Variaciones (EVR) (Izquierdo et al, 2015), which has several potential issues and may give out migration counts which are too low (see Larramona, 2013), it is only available starting with 2008, while the EVR data is available for all years we study. Because of this, we use the age structure of out migration of 2008 obtained from the EM for the years 2004 to 2007. Out migration counts for 2004 to 2007 obtained from the EVR are assumed to be 10% too low and are multiplied by 1.1 to adjust for this issue. This value was obtained from a comparison of the EM and EVR for the years 2008 to 2012.

The approach to correct for migration assumes that out migration does only differ by age, gender, and year and does not differ by occupational category and current state. Unfortunately, we do not have access to data by occupational category. In theory one could use an approach similar as the one outlined above to correct for selectivity regarding inactivity. But while it seems reasonable and is in line with the literature to assume that transitions out of inactivity differ by highest occupational category ever obtained things are not so clear for out migration. While it is known that better educated have a higher probability of emigrating, this somewhat changed during the recession, as lesser educated have been hit more hard than others and economic factors have strong effects on migrating (Izquierdo et al, 2015). Moreover, the population of emigrants is highly selective with respects to other characteristics and consists to a large share of immigrants moving out of Spain (Larramona, 2013). As the CWLS does not contain any information on migration status we stick to the simple approach outlined above. A comparison of corrected and uncorrected results is given below.

A.3. Effects of adjustments

Figure A1 shows a comparison of estimates of WLE by gender and method. Results of the semiparametric method are presented with and without correction, where corrected results are adjusted for selectivity and out migration as outlined above. Survival probabilities

are adjusted using the approach described by Dudel and Myrskylä (2016). Results of the nonparametric approach are also adjusted for out migration. In case of males most estimates are relatively close to each other, except for the year 2007 for which the absolute difference between the nonparametric and the unadjusted semiparametric estimates amounts to 1.9 years. For other years differences are smaller, e.g. 0.8 for 2012. Adjusted semiparametric estimates are mostly close to the nonparametric approach. Correcting for selectivity has an effect on estimates, but this effect is mostly small and differences are only in levels and not in trends. For females the differences between the nonparametric approach and the uncorrected semiparametric approach are larger and range between 2.3 years and 4.4 years. As can be seen in the figure trends are roughly equal but the levels differ considerably. Applying corrections the differences range between 0.0 and 0.6 years. Adjusting transition probabilities thus seems necessary.

Figure A2 shows the effect of adjusting for out migration, showing results based on semiparametric estimates of transition probabilities both unadjusted and adjusted for out migration. For both males and females the effect of adjustment is relatively small for pre-crisis years and the biggest difference between adjusted and unadjusted results amounts to 0.5 years. During the crisis out migration increased considerably which leads to stronger effects of adjustment. For example, for males the difference between adjusted and unadjusted results in 2012 is roughly 1.5 years. Thus, the effect of adjusting for out migration is not as big as the effect of adjusting for sample selectivity, especially for pre-crisis years.

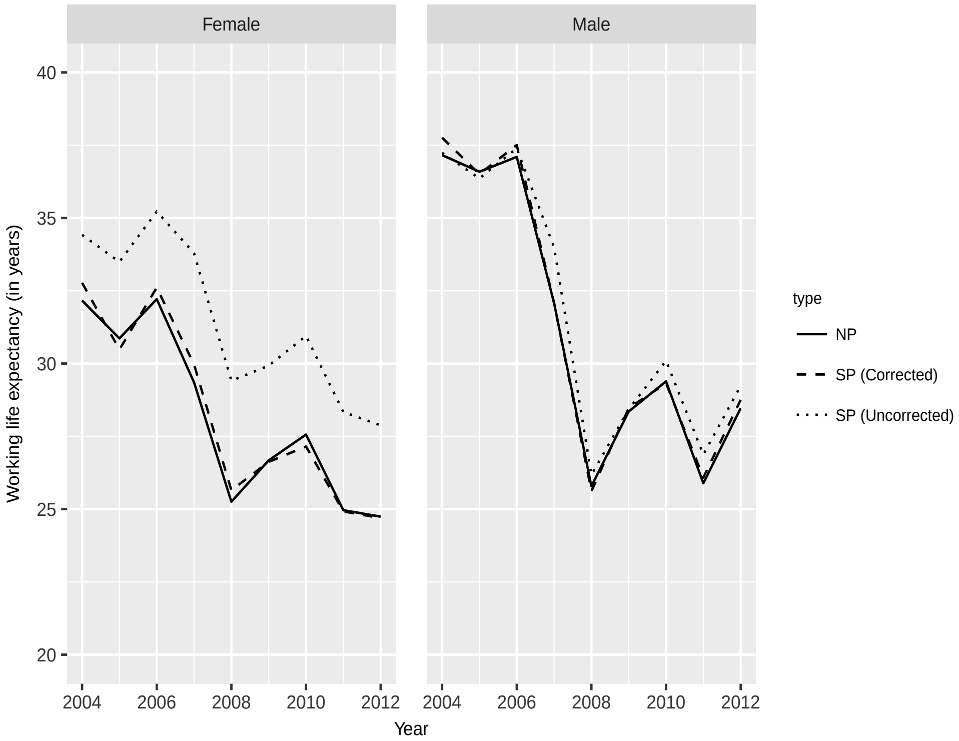

Figure A1: Comparison of WLE estimates over time for males and females by method (NP=Nonparametric; SP=Semiparametric)

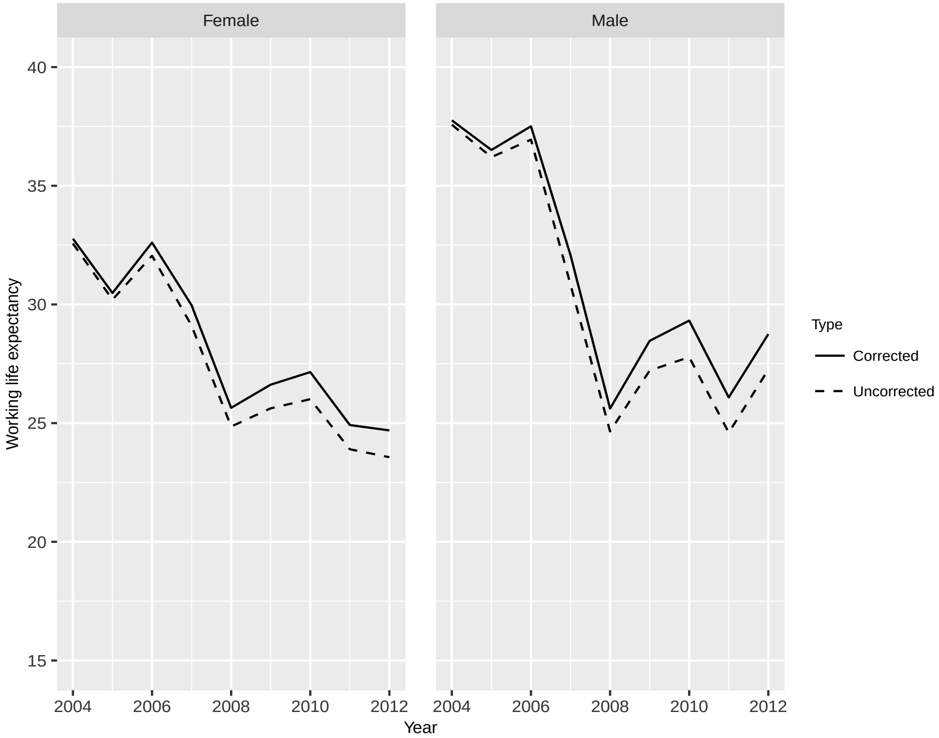

Figure A2: Comparison of WLE estimates adjusted and not adjusted for migration

B. Results of the sensitivity analysis regarding occupational category

As mentioned in the main text in section 3, we are able to assign the highest occupational category ever attained to roughly 91% of the individuals covered by the CWLS. While the occupational category itself changes over time, the highest occupational category ever attained is mostly stable after age 29. For example, between 2004 and 2005 this status is constant for roughly 90% of individuals. To assess whether missing information on occupational category affects our findings, we conducted sensitivity analyses in which all of the individuals with no information on occupational category were classified as skilled non-manual, skilled manual, unskilled non-manual, or unskilled manual. Otherwise, the analyses were carried out as described in the main paper.

Exemplary results of the sensitivity analysis conducted to assess whether missing information on occupational category may bias our results are given in figures B1 and B2. The figures show our original findings for WLE by occupational category from the main text as well as results which were obtained by assuming that individuals with missing occupational category all belong to the category "skilled non-manual". Results of the sensitivity analyses where missing values were replaced with either " skilled manual", "unskilled non-manual", or "unskiled manual" are qualitatively similar.

In all cases levels and trends of WLE obtained from the sensitivity analysis are close to our original analysis. The only exception are skilled non-manual males, for whom the sensitivity analysis shows an increase in WLE from 2004 to 2007. Mean absolute deviations are 0.6 years (skilled non-manual), 0.6 years (skilled manual), 0.4 years (unskilled non-manual), and 0.4 years (unskilled manual) for males. For females mean absolute deviations are all below 0.1 years and results of the analyses are close to identical.

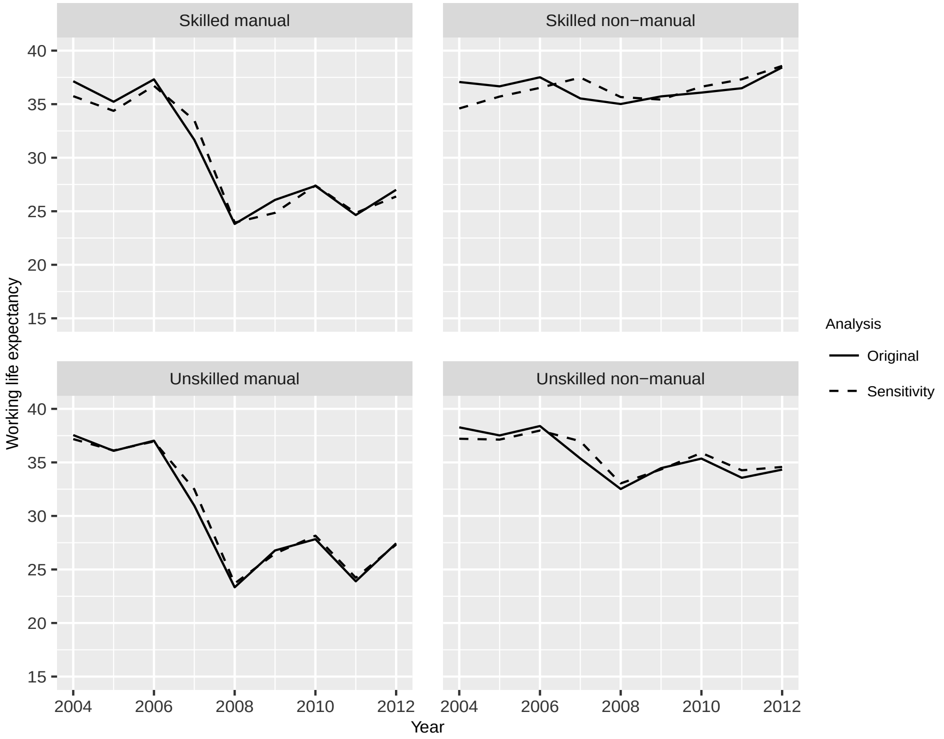

Figure B1: Comparison of WLE estimates from the original and the sensitivity analysis for males.

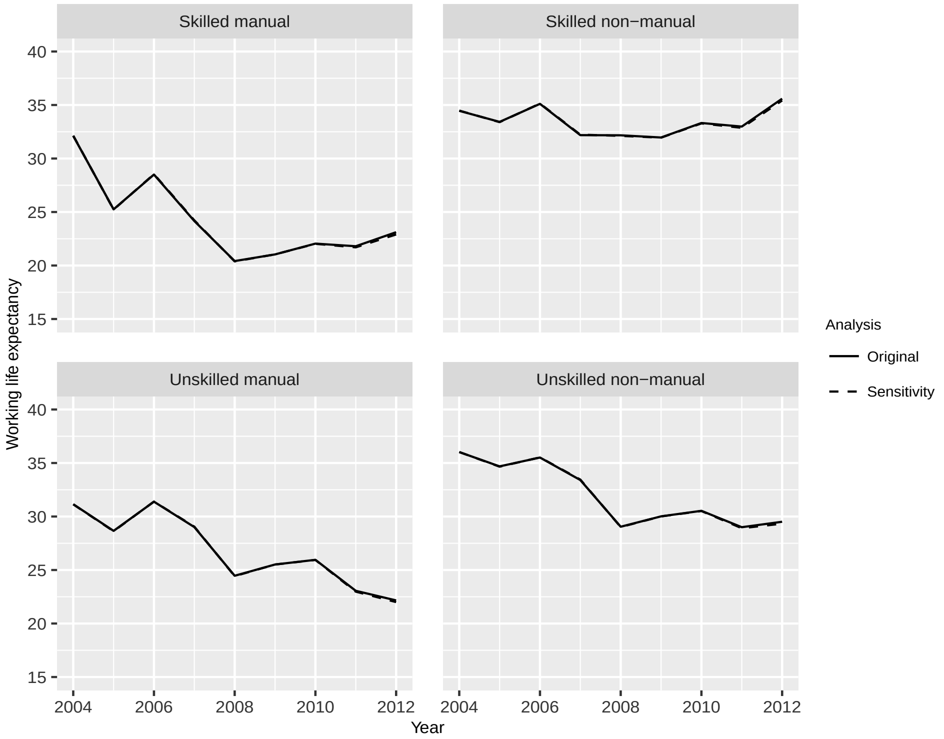

Figure B2: Comparison of WLE estimates from the original and the sensitivity analysis for females.

C. Additional tables

The tables in this section present detailed findings of our analyses.

- Detailed results on life expectancy in all labor force states by gender and occupational category are given in tables C1 to C4. These results can be interpreted as those in table 2 of the main paper and the findings shown in figure 2 of the main paper are extracted from these tables.
- Tables C5 to C8 show detailed results for the composition of working life expectancy by age group discussed in section 4.4 of the main paper. Figure 3 and 4 in the main paper are based on these results.
- Additional results not presented in the main paper are given in tables C9 to C16, which decompose the life expectancy in inactivity and the life expectancy in unemployment by age group, similar to the findings discussed in section 4.4 of the main paper.

Table C1: Life expectancy spent in employment, unemployment, inactivity, and retirement, for Spanish males and females by year; Skilled non-manual

	Employed	Unemployed	Inactive	Retired	Total
Males					
2004/2005	37.07	0.76	9.54	17.04	64.41
2005/2006	36.67	0.65	10.40	16.73	64.44
2006/2007	37.51	0.64	9.79	17.00	64.94
2007/2008	35.53	0.64	12.04	17.66	65.87
2008/2009	35.01	0.89	12.17	17.59	65.66
2009/2010	35.73	1.07	11.13	18.43	66.36
2010/2011	36.08	1.27	10.56	18.44	66.35
2011/2012	36.49	1.69	10.11	19.41	67.69
2012/2013	38.43	1.84	7.82	19.09	67.19
Females					
2004/2005	34.46	0.81	13.10	21.38	69.76
2005/2006	33.42	0.68	14.40	21.54	70.04
2006/2007	35.11	0.72	13.03	21.52	70.38
2007/2008	32.18	0.63	15.82	22.34	70.97
2008/2009	32.17	0.83	16.21	20.72	69.92
2009/2010	31.96	1.04	15.72	21.75	70.47
2010/2011	33.32	1.18	14.74	22.39	71.64
2011/2012	32.98	1.67	14.30	23.21	72.16
2012/2013	35.59	1.97	11.15	21.92	70.63

Table C2: Life expectancy spent in employment, unemployment, inactivity, and retirement, for Spanish males and females by year; Skilled manual

	Employed	Unemployed	Inactive	Retired	Total
Males					
2004/2005	37.14	1.31	6.94	16.84	62.23
2005/2006	35.23	1.19	9.57	16.99	62.97
2006/2007	37.32	1.37	7.67	17.76	64.12
2007/2008	31.67	1.71	13.27	17.06	63.70
2008/2009	23.81	3.07	20.21	17.56	64.65
2009/2010	26.06	3.07	18.01	17.23	64.38
2010/2011	27.37	3.16	16.78	18.14	65.45
2011/2012	24.65	3.58	19.30	17.72	65.24
2012/2013	27.00	4.08	16.30	18.03	65.41
Females					
2004/2005	32.12	2.05	13.59	21.84	69.60
2005/2006	25.25	1.66	21.52	19.99	68.43
2006/2007	28.50	1.90	18.03	20.15	68.58
2007/2008	24.16	1.90	22.14	23.11	71.31
2008/2009	20.42	2.66	25.58	21.98	70.63
2009/2010	21.04	2.94	24.69	20.67	69.34
2010/2011	22.05	2.99	23.86	21.86	70.76
2011/2012	21.80	3.27	23.80	21.82	70.69
2012/2013	23.11	3.50	22.54	21.73	70.88

Table C3: Life expectancy spent in employment, unemployment, inactivity, and retirement, for Spanish males and females by year; Unskilled non-manual

	Employed	Unemployed	Inactive	Retired	Total
Males					
2004/2005	38.27	1.62	6.28	17.34	63.51
2005/2006	37.52	1.41	7.58	16.94	63.46
2006/2007	38.40	1.55	6.80	17.99	64.73
2007/2008	35.37	1.67	9.84	17.77	64.65
2008/2009	32.51	3.04	11.40	17.98	64.94
2009/2010	34.47	3.05	9.57	18.21	65.29
2010/2011	35.36	3.00	8.52	18.15	65.04
2011/2012	33.56	3.41	10.44	18.35	65.76
2012/2013	34.33	3.49	9.30	18.83	65.95
Females					
2004/2005	36.02	2.14	9.68	21.04	68.88
2005/2006	34.69	2.00	11.39	21.13	69.20
2006/2007	35.51	2.14	10.55	21.52	69.72
2007/2008	33.41	2.31	12.51	21.53	69.75
2008/2009	29.04	3.37	16.01	21.43	69.86
2009/2010	30.01	3.60	14.88	22.34	70.82
2010/2011	30.53	3.46	14.52	22.27	70.78
2011/2012	29.00	3.74	15.93	22.05	70.71
2012/2013	29.51	3.88	15.19	22.17	70.75

Table C4: Life expectancy spent in employment, unemployment, inactivity, and retirement, for Spanish males and females by year; Unskilled manual

	Employed	Unemployed	Inactive	Retired	Total
Males					
2004/2005	37.55	3.16	5.09	16.25	62.06
2005/2006	36.08	2.84	7.43	15.64	61.99
2006/2007	37.03	3.40	6.01	15.95	62.39
2007/2008	30.96	5.64	10.09	15.77	62.46
2008/2009	23.34	8.98	14.55	16.02	62.90
2009/2010	26.78	8.62	11.56	16.36	63.32
2010/2011	27.83	7.18	12.12	16.71	63.84
2011/2012	23.90	7.90	15.58	16.54	63.91
2012/2013	27.44	7.31	12.53	16.70	63.98
Females					
2004/2005	31.15	3.84	13.27	20.73	68.99
2005/2006	28.66	3.45	16.64	20.08	68.82
2006/2007	31.39	4.97	12.32	20.76	69.44
2007/2008	29.01	5.81	14.33	19.97	69.12
2008/2009	24.46	7.32	17.95	19.74	69.48
2009/2010	25.52	8.04	15.82	20.16	69.54
2010/2011	25.95	7.02	16.50	20.43	69.91
2011/2012	23.06	6.97	19.28	20.72	70.02
2012/2013	22.16	5.64	21.51	20.76	70.07

Table C5: WLE decomposed by age groups for skilled non-manual males and females

	<19	20-29	30-39	40-49	50-59	60+
Males						
2004/2005	1.13	6.81	9.41	8.99	7.76	2.97
2005/2006	1.24	6.21	9.31	8.96	7.86	3.08
2006/2007	1.34	6.79	9.37	8.98	7.85	3.17
2007/2008	1.20	5.79	8.66	8.93	7.79	3.16
2008/2009	1.15	6.00	8.57	8.69	7.59	3.01
2009/2010	1.22	6.71	8.87	8.68	7.40	2.85
2010/2011	1.34	7.26	8.88	8.54	7.24	2.82
2011/2012	1.41	7.54	8.90	8.47	7.23	2.93
2012/2013	2.02	8.75	9.09	8.39	7.21	2.96
Females						
2004/2005	1.04	6.44	8.44	8.49	7.38	2.68
2005/2006	1.19	6.42	7.92	8.11	7.04	2.73
2006/2007	1.22	6.40	8.08	8.39	7.51	3.51
2007/2008	1.11	5.92	7.55	7.77	6.84	2.98
2008/2009	1.02	6.02	7.51	7.54	6.62	3.46
2009/2010	1.02	6.08	7.80	7.75	6.63	2.68
2010/2011	1.17	6.86	7.98	7.59	6.45	3.27
2011/2012	1.12	6.50	7.89	7.66	6.64	3.19
2012/2013	1.76	8.25	8.45	7.76	6.57	2.80

Table C6: WLE decomposed by age groups for skilled manual males and females

	<19	20-29	30-39	40-49	50-59	60+
Males						
2004/2005	1.57	7.84	9.39	9.04	7.63	1.67
2005/2006	1.51	7.06	9.21	8.69	7.16	1.59
2006/2007	1.71	7.73	9.35	8.98	7.66	1.88
2007/2008	1.32	6.13	8.32	8.08	6.32	1.51
2008/2009	0.86	4.50	6.62	6.30	4.48	1.06
2009/2010	0.96	5.44	7.24	6.61	4.66	1.14
2010/2011	0.97	5.68	7.39	6.90	5.13	1.30
2011/2012	0.86	5.26	6.80	6.14	4.42	1.17
2012/2013	1.08	6.26	7.30	6.42	4.70	1.25
Females						
2004/2005	1.24	7.01	8.15	7.92	6.35	1.45
2005/2006	1.05	5.81	6.48	6.16	4.58	1.18
2006/2007	1.18	6.19	7.08	6.98	5.56	1.50
2007/2008	0.97	5.17	6.15	5.98	4.64	1.26
2008/2009	0.69	4.23	5.30	5.18	3.89	1.11
2009/2010	0.76	4.71	5.66	5.21	3.71	0.99
2010/2011	0.81	5.20	5.84	5.17	3.71	1.31
2011/2012	0.71	4.64	5.77	5.46	4.02	1.21
2012/2013	0.92	5.86	6.18	5.26	3.67	1.23

Table C7: WLE decomposed by age groups for unskilled non-manual males and females

	<19	20-29	30-39	40-49	50-59	60+
Males						
2004/2005	1.69	8.01	9.42	9.14	7.97	2.05
2005/2006	1.79	7.71	9.30	8.90	7.63	2.19
2006/2007	1.82	7.93	9.39	9.07	7.88	2.31
2007/2008	1.50	6.74	8.81	8.75	7.40	2.16
2008/2009	1.14	5.72	8.29	8.39	7.02	1.95
2009/2010	1.31	6.73	8.68	8.48	7.10	2.16
2010/2011	1.47	7.40	8.83	8.56	7.14	1.96
2011/2012	1.26	6.88	8.47	8.15	6.71	2.10
2012/2013	1.42	7.46	8.63	8.14	6.70	1.98
Females						
2004/2005	1.73	8.07	8.62	8.33	7.10	2.18
2005/2006	1.76	7.85	8.23	7.94	6.67	2.24
2006/2007	1.83	7.92	8.32	8.06	6.92	2.47
2007/2008	1.53	7.15	7.98	7.83	6.67	2.25
2008/2009	1.10	6.00	7.08	7.00	5.81	2.05
2009/2010	1.24	6.57	7.41	7.06	5.71	2.04
2010/2011	1.21	6.73	7.54	7.17	5.80	2.08
2011/2012	1.08	6.23	7.16	6.85	5.56	2.11
2012/2013	1.21	6.90	7.42	6.83	5.34	1.82

Table C8: WLE decomposed by age groups for unskilled manual males and females

	<19	20-29	30-39	40-49	50-59	60+
Males						
2004/2005	2.28	8.48	9.04	8.67	7.28	1.81
2005/2006	2.24	8.27	8.91	8.26	6.68	1.72
2006/2007	2.34	8.40	8.97	8.47	6.99	1.86
2007/2008	1.79	6.92	7.94	7.33	5.54	1.44
2008/2009	1.16	5.11	6.34	5.77	4.02	0.93
2009/2010	1.35	6.04	7.04	6.48	4.69	1.18
2010/2011	1.25	6.16	7.28	6.75	5.03	1.36
2011/2012	0.99	5.43	6.55	5.89	4.08	0.96
2012/2013	1.14	6.16	7.25	6.63	4.92	1.33
Females						
2004/2005	1.82	7.73	7.66	7.04	5.46	1.44
2005/2006	1.74	7.42	7.13	6.37	4.74	1.25
2006/2007	1.83	7.50	7.39	6.95	5.62	2.10
2007/2008	1.54	6.65	6.83	6.45	5.14	2.40
2008/2009	1.09	5.38	5.73	5.43	4.25	2.59
2009/2010	1.27	5.94	6.15	5.76	4.36	2.03
2010/2011	1.17	6.02	6.35	5.92	4.48	2.01
2011/2012	0.93	5.21	5.87	5.57	4.08	1.41
2012/2013	1.01	5.81	5.94	5.18	3.47	0.74

Table C9: Inactive life expectancy decomposed by age groups for skilled non-manual males and females

	<19	20-29	30-39	40-49	50-59	60+
Males						
2004/2005	3.86	3.04	0.36	0.62	1.27	0.88
2005/2006	3.75	3.65	0.50	0.71	1.34	0.95
2006/2007	3.65	3.06	0.44	0.70	1.36	1.07
2007/2008	3.79	4.11	1.16	0.79	1.57	1.13
2008/2009	3.84	3.81	1.19	0.98	1.71	1.14
2009/2010	3.77	3.05	0.86	0.95	1.81	1.21
2010/2011	3.64	2.45	0.81	1.06	1.90	1.20
2011/2012	3.57	2.16	0.71	1.06	1.89	1.21
2012/2013	2.94	0.84	0.47	1.08	1.90	1.08
Females						
2004/2005	3.95	3.37	1.36	1.26	2.05	1.61
2005/2006	3.80	3.43	1.90	1.64	2.41	1.72
2006/2007	3.77	3.46	1.76	1.38	1.92	1.24
2007/2008	3.88	3.94	2.28	1.99	2.62	1.61
2008/2009	3.96	3.76	2.25	2.18	2.84	1.71
2009/2010	3.97	3.63	1.94	1.97	2.88	1.84
2010/2011	3.81	2.76	1.69	2.09	3.03	1.87
2011/2012	3.86	2.97	1.68	1.95	2.74	1.60
2012/2013	3.20	1.12	1.00	1.77	2.78	1.77

Table C10: Inactive life expectancy decomposed by age groups for skilled manual males and females

	<19	20-29	30-39	40-49	50-59	60+
Males						
2004/2005	3.41	1.90	0.24	0.41	0.87	0.60
2005/2006	3.47	2.71	0.46	0.82	1.52	1.09
2006/2007	3.27	2.02	0.30	0.53	1.09	0.95
2007/2008	3.66	3.57	1.25	1.31	2.36	1.63
2008/2009	4.11	5.00	2.60	2.74	3.89	2.36
2009/2010	4.01	3.99	1.99	2.50	3.78	2.25
2010/2011	4.01	3.80	1.85	2.20	3.34	2.08
2011/2012	4.11	4.14	2.31	2.85	3.99	2.40
2012/2013	3.89	3.06	1.70	2.44	3.60	2.12
Females						
2004/2005	3.75	2.67	1.34	1.54	2.56	2.23
2005/2006	3.94	3.95	3.09	3.38	4.55	3.11
2006/2007	3.80	3.58	2.48	2.53	3.46	2.67
2007/2008	4.02	4.53	3.37	3.48	4.36	2.87
2008/2009	4.29	5.39	4.03	4.11	4.98	3.28
2009/2010	4.22	4.83	3.55	4.00	5.15	3.44
2010/2011	4.17	4.26	3.33	4.07	5.21	3.33
2011/2012	4.27	4.87	3.41	3.71	4.77	3.26
2012/2013	4.06	3.54	2.85	3.87	5.24	3.48

Table C11: Inactive life expectancy decomposed by age groups for unskilled non-manual males and females

	<19	20-29	30-39	40-49	50-59	60+
Males						
2004/2005	3.28	1.66	0.21	0.33	0.74	0.56
2005/2006	3.19	1.99	0.33	0.59	1.13	0.85
2006/2007	3.16	1.76	0.25	0.44	0.91	0.79
2007/2008	3.47	2.93	0.78	0.70	1.40	1.06
2008/2009	3.82	3.72	1.03	0.79	1.44	1.10
2009/2010	3.65	2.62	0.63	0.71	1.43	1.03
2010/2011	3.49	2.02	0.49	0.65	1.34	1.03
2011/2012	3.71	2.58	0.76	0.95	1.73	1.21
2012/2013	3.54	2.00	0.58	0.91	1.67	1.10
Females						
2004/2005	3.24	1.48	0.86	1.15	1.92	1.53
2005/2006	3.21	1.74	1.27	1.54	2.36	1.78
2006/2007	3.14	1.65	1.17	1.40	2.07	1.62
2007/2008	3.45	2.39	1.46	1.60	2.31	1.80
2008/2009	3.87	3.33	2.08	2.19	2.95	2.09
2009/2010	3.73	2.70	1.69	2.11	3.04	2.12
2010/2011	3.76	2.58	1.59	2.03	2.97	2.09
2011/2012	3.89	3.09	1.87	2.24	3.15	2.19
2012/2013	3.75	2.40	1.57	2.25	3.38	2.35

Table C12: Inactive life expectancy decomposed by age groups for unskilled manual males and females

	<19	20-29	30-39	40-49	50-59	60+
Males						
2004/2005	2.66	0.80	0.20	0.39	0.86	0.68
2005/2006	2.70	1.08	0.37	0.87	1.63	1.27
2006/2007	2.60	0.84	0.25	0.60	1.18	1.04
2007/2008	3.12	1.90	0.73	1.20	2.11	1.53
2008/2009	3.75	3.29	1.51	1.85	2.73	1.93
2009/2010	3.54	2.22	0.92	1.37	2.29	1.72
2010/2011	3.67	2.48	1.04	1.42	2.31	1.71
2011/2012	3.95	3.39	1.61	2.03	2.97	2.11
2012/2013	3.79	2.71	1.04	1.41	2.32	1.75
Females						
2004/2005	3.13	1.27	1.29	2.08	3.29	2.71
2005/2006	3.21	1.71	1.93	2.83	4.16	3.30
2006/2007	3.11	1.56	1.46	2.02	2.74	1.92
2007/2008	3.39	2.20	1.78	2.34	3.06	2.05
2008/2009	3.84	3.13	2.39	2.93	3.67	2.49
2009/2010	3.65	2.34	1.75	2.46	3.42	2.71
2010/2011	3.77	2.55	1.84	2.54	3.55	2.76
2011/2012	4.02	3.55	2.37	2.85	3.88	3.10
2012/2013	3.94	2.99	2.40	3.41	5.02	4.24

Table C13: Life expectancy in unemployment decomposed by age groups for skilled non-manual males and females

	<19	20-29	30-39	40-49	50-59	60+
Males						
2004/2005	0.01	0.11	0.13	0.13	0.24	0.14
2005/2006	0.01	0.09	0.10	0.10	0.21	0.14
2006/2007	0.01	0.10	0.09	0.09	0.20	0.15
2007/2008	0.00	0.07	0.10	0.12	0.21	0.13
2008/2009	0.01	0.16	0.17	0.17	0.26	0.12
2009/2010	0.01	0.20	0.19	0.19	0.31	0.16
2010/2011	0.02	0.26	0.23	0.23	0.36	0.18
2011/2012	0.02	0.27	0.34	0.35	0.49	0.22
2012/2013	0.03	0.38	0.39	0.40	0.48	0.15
Females						
2004/2005	0.01	0.18	0.16	0.14	0.22	0.11
2005/2006	0.01	0.13	0.13	0.12	0.19	0.09
2006/2007	0.01	0.13	0.13	0.12	0.21	0.13
2007/2008	0.01	0.12	0.13	0.12	0.17	0.08
2008/2009	0.01	0.20	0.19	0.16	0.20	0.07
2009/2010	0.01	0.28	0.24	0.20	0.24	0.07
2010/2011	0.02	0.36	0.29	0.22	0.23	0.06
2011/2012	0.02	0.52	0.40	0.31	0.33	0.08
2012/2013	0.04	0.61	0.50	0.39	0.37	0.07

Table C14: Life expectancy in unemployment decomposed by age groups for skilled manual males and females

	<19	20-29	30-39	40-49	50-59	60+
Males						
2004/2005	0.01	0.19	0.20	0.20	0.46	0.24
2005/2006	0.01	0.18	0.20	0.20	0.41	0.19
2006/2007	0.02	0.20	0.23	0.23	0.46	0.23
2007/2008	0.02	0.27	0.34	0.36	0.53	0.21
2008/2009	0.03	0.45	0.67	0.72	0.89	0.31
2009/2010	0.03	0.53	0.66	0.66	0.85	0.32
2010/2011	0.03	0.49	0.70	0.73	0.91	0.29
2011/2012	0.03	0.57	0.80	0.84	1.01	0.33
2012/2013	0.03	0.65	0.92	0.97	1.14	0.35
Females						
2004/2005	0.01	0.30	0.45	0.39	0.63	0.27
2005/2006	0.01	0.22	0.36	0.32	0.50	0.26
2006/2007	0.01	0.22	0.38	0.36	0.60	0.32
2007/2008	0.01	0.28	0.42	0.38	0.56	0.24
2008/2009	0.02	0.36	0.62	0.59	0.77	0.31
2009/2010	0.02	0.44	0.73	0.66	0.79	0.29
2010/2011	0.02	0.51	0.75	0.63	0.76	0.31
2011/2012	0.02	0.48	0.79	0.72	0.89	0.38
2012/2013	0.02	0.58	0.94	0.79	0.84	0.32

Table C15: Life expectancy in unemployment decomposed by age groups for unskilled non-manual males and females

	<19	20-29	30-39	40-49	50-59	60+
Males						
2004/2005	0.02	0.30	0.28	0.27	0.51	0.24
2005/2006	0.02	0.25	0.25	0.24	0.44	0.21
2006/2007	0.02	0.27	0.25	0.25	0.49	0.26
2007/2008	0.02	0.28	0.30	0.31	0.51	0.25
2008/2009	0.03	0.52	0.59	0.62	0.92	0.36
2009/2010	0.04	0.61	0.61	0.61	0.86	0.33
2010/2011	0.03	0.55	0.60	0.61	0.88	0.33
2011/2012	0.03	0.50	0.69	0.73	1.04	0.42
2012/2013	0.03	0.52	0.74	0.80	1.05	0.35
Females						
2004/2005	0.02	0.43	0.48	0.40	0.58	0.23
2005/2006	0.02	0.39	0.46	0.38	0.53	0.21
2006/2007	0.03	0.41	0.45	0.39	0.59	0.28
2007/2008	0.03	0.44	0.52	0.45	0.63	0.25
2008/2009	0.03	0.65	0.80	0.70	0.89	0.32
2009/2010	0.03	0.72	0.87	0.73	0.91	0.33
2010/2011	0.03	0.68	0.83	0.70	0.89	0.34
2011/2012	0.03	0.66	0.92	0.80	0.97	0.35
2012/2013	0.03	0.70	0.98	0.84	0.97	0.35

Table C16: Life expectancy in unemployment decomposed by age groups for unskilled manual males and females

	<19	20-29	30-39	40-49	50-59	60+
Males						
2004/2005	0.06	0.66	0.60	0.59	0.91	0.35
2005/2006	0.05	0.59	0.56	0.54	0.79	0.31
2006/2007	0.06	0.70	0.64	0.63	0.96	0.41
2007/2008	0.08	1.13	1.19	1.17	1.50	0.55
2008/2009	0.09	1.54	2.03	2.09	2.43	0.80
2009/2010	0.10	1.70	1.93	1.90	2.25	0.72
2010/2011	0.07	1.33	1.59	1.61	1.94	0.64
2011/2012	0.06	1.15	1.75	1.85	2.24	0.84
2012/2013	0.06	1.09	1.62	1.75	2.08	0.70
Females						
2004/2005	0.06	0.97	0.99	0.74	0.86	0.23
2005/2006	0.05	0.85	0.90	0.67	0.77	0.22
2006/2007	0.06	0.92	1.11	0.92	1.31	0.66
2007/2008	0.06	1.13	1.35	1.10	1.47	0.70
2008/2009	0.07	1.47	1.84	1.52	1.76	0.66
2009/2010	0.08	1.70	2.05	1.67	1.89	0.66
2010/2011	0.07	1.42	1.77	1.45	1.68	0.64
2011/2012	0.05	1.23	1.73	1.48	1.74	0.74
2012/2013	0.05	1.18	1.61	1.30	1.22	0.27

References

Dudel C, Myrskylä M (2016) Recent trends in US working life expectancy by sex, education, and race and the impact of the Great Recession, MPIDR Working Paper WP-2016-06

Human Mortality Database (2015) University of California, Berkeley (USA), and Max Planck Institute for Demographic Research (Germany), available at www.mortality.org or www.humanmortality.de. Accessed 30 July 2016

Izquierdo M, Jimeno JF, Lacuesta A (2015) Spain: From immigration to emigration?, Banco de Espana Working Paper 1503

Larramona G (2013) Out-migration of immigrants in spain. Population 68:249–271
